# The role of the user within the medical device design and development process: medical device manufacturers' perspectives

**DOI:** 10.1186/1472-6947-11-15

**Published:** 2011-02-28

**Authors:** Arthur G Money, Julie Barnett, Jasna Kuljis, Michael P Craven, Jennifer L Martin, Terry Young

**Affiliations:** 1 Department of Computer Science and Technology, University of Bedfordshire, Park Square, Luton, LU1 3JU, UK; 2 Department of Information Systems and Computing, Brunel University, Uxbridge, UB8 3PH, UK; 3 Department of Electrical and Electronic Engineering, University of Nottingham, University Park, Nottingham, NG7 2RD, UK

## Abstract

**Background:**

Academic literature and international standards bodies suggest that user involvement, via the incorporation of human factors engineering methods within the medical device design and development (MDDD) process, offer many benefits that enable the development of safer and more usable medical devices that are better suited to users' needs. However, little research has been carried out to explore medical device manufacturers' beliefs and attitudes towards user involvement within this process, or indeed what value they believe can be added by doing so.

**Methods:**

In-depth interviews with representatives from 11 medical device manufacturers are carried out. We ask them to specify who they believe the intended users of the device to be, who they consult to inform the MDDD process, what role they believe the user plays within this process, and what value (if any) they believe users add. Thematic analysis is used to analyse the fully transcribed interview data, to gain insight into medical device manufacturers' beliefs and attitudes towards user involvement within the MDDD process.

**Results:**

A number of high-level themes emerged, relating who the user is perceived to be, the methods used, the perceived value and barriers to user involvement, and the nature of user contributions. The findings reveal that despite standards agencies and academic literature offering strong support for the employment formal methods, manufacturers are still hesitant due to a range of factors including: perceived barriers to obtaining ethical approval; the speed at which such activity may be carried out; the belief that there is no need given the 'all-knowing' nature of senior health care staff and clinical champions; a belief that effective results are achievable by consulting a minimal number of champions. Furthermore, less senior health care practitioners and patients were rarely seen as being able to provide valuable input into the process.

**Conclusions:**

Medical device manufacturers often do not see the benefit of employing formal human factors engineering methods within the MDDD process. Research is required to better understand the day-to-day requirements of manufacturers within this sector. The development of new or adapted methods may be required if user involvement is to be fully realised.

## Background

From a human factors engineering perspective, ensuring the development of high quality and well designed medical devices that are in tune with patient and user needs, require formal human factors engineering methods (also known as user-centred usability engineering methods) to be used at every stage of the MDDD process [1]. Employing such formal methods ensures that the device design process considers appropriately the environment in which the device is to be used, the work patterns of users and the specific individual needs of the user, which could be any individual involved in the use of the device including health professionals, patients, and lay care givers [2]. In particular, human factors engineering methods highlight the importance of considering the needs of the user when designing and developing devices at the earliest stage of defining the device concept and then at every subsequent stage of the device development process [3]. The importance and value of focusing on user needs has been recognised as having a number of health related benefits including; improved patient safety [4,5], improved compliance and health outcomes [6], and higher levels of patient and user satisfaction [7]. Furthermore, employing human factors engineering methods throughout the MDDD process has been said to substantially reduce device development time because usability issues are identified and attended to prior launch, and hence avoid costly design changes and product recalls [8,9].

Given the proposed benefits of developing medical devices according to a human factors engineering perspective, medical device standards bodies are increasingly recognising the importance of these methods and the important role they play in developing safe and usable medical devices. Currently the two most important regulations for medical device developers are the European Commission (EC) Medical Device Directive 93/42/EEC [10] and the United States (US) Food and Drug Administration (FDA) regulations since medical devices must comply with these regulations in order to be sold throughout Europe and the US [11]. These regulations promote the use of human factors engineering methods within the MDDD process, for example, the US FDA regulations specify that medical device developers must demonstrate that human factors principles have been used in the design of the device so as to ensure that use-related hazards have been identified, understood and addressed. Some guidance is also given suggesting how human factors principles may be integrated into the MDDD process [12]. A further standard that medical device developers have been obliged to adhere in recent years is the International Electrotechnical Commission (IEC) standard 60601-1-6 [13], which have equivalent European Standards (ES) and British Standards (BS), requiring medical device developers to incorporate human factors engineering processes to ensure patient safety, stating that "The manufacturer should conduct iterative design and development. Usability engineering should begin early and continue through the equipment design and development lifecycle". More recently, the usability standard 'IEC 62366: Medical devices - Application of usability engineering to medical devices' [14] has superseded IEC 60601-1-6, and extends the requirement for medical device developers to incorporate human factors engineering methods in the development of all medical devices, not just electrical devices. In early 2010 IEC 62366 was harmonised by the EU Medical Device Directive meaning that it is now a legal requirement for medical device developers to formally address the usability of a device before placing it on the market anywhere in Europe. In order to comply with this standard, medical device developers must document the process in detail within a Usability Engineering File.

### Human Factors Engineering Methods

The MDDD process may be considered to be made up of four key stages. At stage one, user needs are established and scoping exercises are carried out with users. Stage two aims to validate and refine the concept. Stage three involves designing the device, and Stage four involved evaluation of the device.

**Figure 1:**
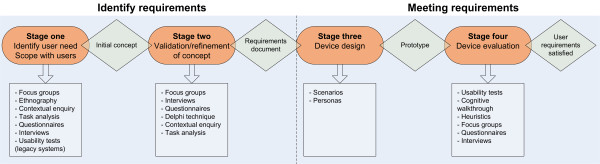
**Medical device development lifecycle adapted from **[5].

Figure 1 presents the medical device development lifecycle, and the associated user-centred design methods that may be used at each respective stage.

In some of our earlier work, as part of the Multidisciplinary Assessment of Technology Centre for Healthcare (MATCH) research activity, we carried out a rigorous review of the methods that have been employed in the MDDD process as presented in the academic literature [5,11,15]. MATCH is a UK research initiative between five universities which is funded by the Engineering and Physical Sciences Research Council (EPSRC) and the Department of Trade and Industry (DTI). The aim of MATCH is to provide support to the healthcare sector by developing methods to assess the value of medical devices at every stage of the MDDD process, with a focus on working with industrial partners to solve real world problems.

To provide some insight into what a selection of these methods involve, a brief description of the most frequently occurring methods within the device development lifecycle are now provided. For a detailed description of all methods and how these may be applied within the MDDD is provided in [5].

*Focus groups *involve group discussion typically between 8-10 users, with a moderator guiding and facilitating the group through relevant topics of discussion. They may be appropriate for use at stage one, two or four of the device development lifecycle. Focus groups are used in a wide variety of industry settings and may be conducted at a comparatively low cost compared with other methods such as usability testing.

*Interviews *are one of the most common approaches employed in user centred research, and may be of value at stages one, two and four of the development pathway. They can be rapidly deployed, and are relatively inexpensive to carry out. Interviews enable the researcher to access a broad range of opinion regarding a device, whilst also allowing rich and detailed opinions to be collected.

*Usability testing*, typically performed at stage four, advocates is a function of three criteria: effectiveness, efficiency and satisfaction. Usability testing protocols involve users carrying out specific tasks with a specific device, whilst usability measures are taken. Effectiveness is concerned with whether the user is able to successfully accomplish a given task. Efficiency may include a count of the number of user actions or the amount of time required to carry out a task. Satisfaction may typically be measured subjectively by means of a satisfaction questionnaire, completed after using a particular device.

*Heuristic evaluation *is a more rapid form of usability test which may be deployed by developers, perhaps prior to carrying out usability tests. Developers step through the device features and functionality and check the extent to which it complies with pre-determined list of characteristics (heuristics/rules of thumb) that may have been defined by earlier UCD design activities. This type of evaluation would normally be applied at stage four of the development pathway, once a tangible device has been developed.

### The challenge for industry

Although a large number of human factors engineering methods are available that may be employed within the MDDD process, previous research indicates that medical device manufacturers often avoid employing such methods due to lack of resources and the perception that such methods are often too resource intensive [16]. The literature in the area of user involvement suggests that there are a number of risks for manufacturers associate with a lack of engagement with users within the product development process. For example, Cooper [17] suggests that failure to build in the voice of the customer is one of the key reasons for the failure of developing effective and innovative products. Certainly in the realm of ICT products Kujala [18] suggests that attending to user perspectives increase the likelihood of a successful product. Alongside this however, other work has highlighted some of the challenges of, and barriers to, involving users in the product development process. Brown [19] and Butler [20] both suggest that the volume of data generated by field studies with users may be costly, difficult to analyse with no obvious route to informing development. When considering the factors leading to successful innovation, van der Panne et al. [21] note that although it is incontrovertible that good market research is associated with a successful product, the role of customer involvement in innovation remains contentious. They suggest that customer involvement at an early stage may tend to gravitate towards imitative products, being less able to envision or express novelty and thus, "bias innovation efforts towards incremental innovation" [[21] p.326]. User preferences may change over time and engaging with a limited range of users may result in over specification of the product [22]. From the developer's perspective, their criteria for the success of user involvement may be different than those (often academics or researchers) who actually *do *user engagement work [23,24]. Furthermore, user information that is based on formal methods of elicitation may be at variance with the representations of the user held by developers themselves [22] and may thus not readily be appropriated within the organisational culture and structure of the lead organisation [25]. Many of the above examples are drawn from the more general area of user involvement within the product development process, since there is limited research in this area specifically addressing such issues within the medical device development domain. There is a lack of existing primary research that explores the challenges and benefits of involving users specifically within the medical device development process, particularly from a medical device manufacturer's perspective. Examples of some of the work that does exist within this domain includes, Grocott et al. [26] who carry out pioneering work in the field, proposing a valuable model of user engagement in medical device development, and focus on the practical issues around how user needs may be captured throughout the MDDD process [26]. Shah and Robinson [16] carry out secondary research in the form of a literature review of research that has involved users in some way in the design and development of medical devices, exploring the barriers and benefits to involving users as may have been reported in the reviewed literature. The findings of this study are in line with the perspective offered above that whilst user involvement in MDDD process is likely to benefit the user in terms of developing devices better suited to the users' needs, MDDD methods may be perceived as highly resource intensive and hence often not a feasible option for some manufacturers.

Reflecting on the body of literature above, and in particular on the perceived benefits and barriers to user involvement within the wider domain of technology product development, it seems that there are a number of key factors that impact on effective engagement with users. Perhaps most notable is the notion that product developers consider user needs research to be disproportionately costly, in light of the perceived benefits and pay-off for engaging in such activities. Furthermore, user needs research is perceived by developers to generate unmanageable volumes of data and there does not appear to be any clear route through to informing the development of the product once the user needs data has been collected and analysed. Whilst all of the perceived drawbacks, if well-founded, may be notable reasons for avoiding engagement with user needs research, it is not clear what the underlying reasons are for these factors. For example, a commonly held view by some developers is that the key function of human factors engineering methods is to serve as a means of facilitating a 'cake-frosting' exercise [27], by which 'superficial' design features may be 'painted' onto the device at the end of the development process. Such views of human factors engineering methods do not lend themselves to positive engagement or indeed a realisation of the full complexity and pay-off that such methods may potentially deliver if they were to be deployed with methodological rigour at appropriate points within the design process [28]. Certainly from an academic and/or human factors engineer's point of view, it may be argued that many of these factors may be overcome by increased awareness and better educating industrial developers in human factors engineering methods and their application. However, is it the case that with more training in the area of user research methods research, product developers may overcome these reservations, recognise the potential opportunities these methods promise, and develop the necessary skills to deploy methods and analyse data in a timely fashion, or are these methods indeed incompatible within the industrial context? Should user needs research be outsourced and delivered by the human factors engineering experts, in order to overcome the overhead in acquiring appropriate skills and level of understanding to actually effectively deploy these methods? Should these methods be adapted to make them more lightweight and easier to implement, hence making them more fit for purpose? These are all questions that need to be answered if the goal of incorporating the user into the product development process is to be realised. More specifically within the MDDD process, there are likely to be domain specific factors and conditions that influence the uptake of such methods.

Therefore, from a theoretical perspective, human factors engineering methods are presented as being of value at every stage of the MDDD process, manufacturers may not actually be employing these methods in practice. If their full benefits are to be realised, more primary research is needed to better understand manufacturers' perspectives and motivations regarding such methods. Therefore, the aim of this study is to gain first hand and detailed insights into what medical device manufacturers' attitudes are towards engaging with users, and what the perceived value and barriers are of doing so. Furthermore, we aim to explore which methods are used, and what device manufacturers' attitudes are towards employing such methods.

### Section summary

Thus far, we have detailed a range of formal methods that may be used to elicit user perspectives as part of the process of medical device development and noted the range of regulatory requirements to involve users. We have suggested that the 'in principle' value of systematically seeking user input may be somewhat at variance with the day to day experiences of manufacturers where user involvement may be seen as a barrier to speedy and innovative product development. There is currently no evidence about this in respect of medical device development. The remainder of this paper addresses this issue and is structured as follows. In the next section, we describe a study which involved carrying out in-depth interviews with 11 medical device manufacturers which explored the perceived value of users in the MDDD process. In the following section, the results of the analysis of these interviews are then presented. The paper concludes with suggested recommendations for future research and practice in this area.

## Methods

Eleven interviews were carried out with senior members of staff in each company. Recruitment was by convenience sampling: participants were identified as a result of their employment by companies that were industrial partners of the MATCH collaboration. To provide case examples for discussion, company representatives were asked to choose one medical device to discuss during the interview, which provided an example of the MDDD process they engaged in. The majority of the devices chosen for discussion were intended for use by surgeons within a clinical setting. Interviews lasted approximately one hour in duration, and were carried out by members of the MATCH research team. One of the topics of discussion was around device users. Interviewees were asked about the role the user is perceived to play within the design and development of the example medical device, what they saw as the barriers and benefits to involving users within the MDDD process, and what human factors engineering methods they used (if any) within the MDDD process. All interviews were recorded and researchers also took notes during the interviews. Before beginning, the interviewer explained the purpose and format of the interview to the participant and informed consent to participate, and for the audio recording of the interview, was obtained. Table 1 summarises the position held within the company for each interviewee, the treatment area or clinical use for the device, the intended users of the devices, and the actual individuals consulted in the MDDD process by the respective manufacturers.

**Table 1:** Company details and intended device users

	Position held	Device's treatment area or clinical use	Intended users	Individuals consulted
**#1**	Operations Mgr & Clin. Affairs Mgr.	Orthopaedics	Surgeons	Surgeons

**#2**	Operations Mgr	Cardiology	Health profs. General public	Health profs.

**#3**	Scientific Director	Oncology	Health profs. Physicians	Health profs. Trainee health professionals

**#4**	Director	Spectroscopy	Health profs. Clinicians	Electronics experts

**#5**	Director of Clin. Research & Clin. Reimbursement Mgr.	Orthopaedics	Surgeons	Surgeons

**#6**	Managing Director	Orthopaedics	Surgeons	Surgeons

**#7**	R&D Mgr.	Orthopaedics	Surgeons	Surgeons

**#8**	R&D Director	Laryngology	Gen. health practitioners	Purchasing reps. Clinical champion

**#9**	Managing Director	Phlebotomy	Clinicians University researchers	Clinicians University researchers

**#10**	Medical Tech. Mgr.	Vital signs monitoring	Nurses Clinicians	Physicians

**#11**	Research Programme Mgr.	Wound care	Home patients	Surgeons

**Figure 2:**
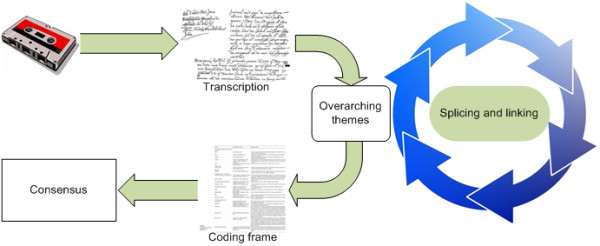
**Process of thematic analysis**.

A thematic analysis of the textual dataset was then carried out. Detailed descriptions of what the thematic analysis process involves are available in [29-32]. In brief, thematic analysis facilitates the effective and rigorous abstraction of salient themes and sub-themes from a complex and detailed textual dataset [33], hence is particularly suitable in this context. The following steps were taken to analyse the data collected during the interview process, Figure 2 provides an overview of this.

Initially all recordings of the trial sessions were transcribed into text format. After transcription, the textual dataset was initially perused to conceptualise the overarching themes that existed within the transcripts at a high-level. These were noted in a coding frame, with each concept assigned a code name, and a description and examples of text that fit each concept. The dataset was then examined iteratively, enabling themes and sub-themes to be developed further. These were spliced and linked together, and text relating to each category and sub-category were appropriately labelled. The first and second authors coded the data and discussed inconsistencies where these arose until a clear consensus of the main themes was reached. When no further refinement of the categorisation could be derived a final group of categories and sub-categories, representative of the transcripts was produced. The main themes are those drawn from multiple contributions and that represent issues that are clearly central to the participants themselves. Within these themes, we have explored areas of consensus and diversity as they were presented by interviewees.

## Results

As a result of carrying out a thematic analysis of the interviews carried out with manufacturers, four high level themes emerged relating to manufacturers' views of user involvement within the MDDD process. These are as follows: Who is the user?; Methods used; Perceived value and barriers to user involvement; The nature of user contributions. The remainder of this section presents the findings of our study according to these themes.

### Who is the User?

Discussions relating to the range of individuals that are consulted during the MDDD process revealed that there was a mismatch between the users that were consulted, and those that would actually use the device in practice. Some manufacturers believed that the needs of the patient do not originate from the patient themselves, and that patients' needs are better articulated through a hierarchy of health professionals including surgeons and 'clinical champions'.

**#2**: "The need actually will probably be established by the clinical fraternity....All you have to do after that is convince the people on down the chain, from the hospital clinical researchers right down to the guy in the street who says 'It's a good idea to have one of these. That's where the need is identified. It is identified really at the top, and then it's taught down, if you follow me"

In light of this, manufacturers had a preference for seeking input from more senior health care staff over less senior staff, regardless of who would actually use the device in practice. The assumption was that senior staff members were more than capable of speaking on behalf of less senior staff, even though manufacturers acknowledged that it was likely that senior staff members would never actually use the device. This was the case even in scenarios where the patient was seen to be the main user of the device. For example, Manufacturer #2 who is involved in the development of Automated External Defibrillators (AEDs), recognised that a significant proportion of the intended users of the device included members of the public, however, they did not see it as necessary to consult members of the public, but rather consulted senior health professionals in the early stages of device design and development. Input from nurses was also considered to be less desirable than input from more senior health care staff such as surgeons. For example, Manufacturer #11 identified nurses as the main user of their product, however, did not consider it necessary to consult nurses in the design and development process. It was felt that surgeons made the decisions on behalf of nurses and patients, and therefore it was logical to consult them to identify nurse and patient needs, as is articulated below.

**#11**: "Surgeons may be there making the decision or recommending which models to buy, but it might be the nurses who are actually using the unit...the surgeon has made the decision, but he doesn't necessarily have to actually work with it, so in your case, the controllability aspect, the patient actually understanding how to operate the unit, the clinician has made the decision and has prescribed that particular device..."

Surgeons were also considered by Manufacturer #11 as having sufficient knowledge to act as representatives for home patients. The motivation for this was as a direct result of the way in which the device is introduced and promoted to the patient. Surgeons do the marketing and promoting of the product to the patients, and therefore it was seen as important that the device is primarily designed according to the surgeons' requirements. In the quote below, manufacturer #11 justifies their motivation for valuing the surgeons' opinion over the patient users.

**#11**: "but, by and large, most of our market research is done with the surgeons, not with the end users, rightly or wrongly...but how it will be marketed will be through the healthcare professional, who will have to sell it effectively to the patient, show them how to use it."

Similarly, in the event of the user group being general health professionals, the opinions of a small number of 'clinical champions' was sought by Manufacturer #8 as well as purchasing representatives from the health authority. It was deemed more important to meet the needs of the individuals who were responsible for making purchasing decisions or held most influence over them, as opposed to focusing on the needs of the individuals that would be using the devices on a day to day basis. Therefore MDDD activity often seemed to be carried out a strong focus on how effort would translate to sales. In the following quote, Manufacturer #4 is asked who would be consulted in identifying design requirements for the development of the given medical device:

**#4: P**^**1**^: "...it's through the doctors to, its [name]'s contact with the hospitals out there, now then I doubt it would be at doctor level, it would be more the managerial level of the hospital..."

**I**^**2**^: ...so in effect you are capturing a user need in that way...

**P**: " ...yes."

(Note: ^1 ^***P: ***Denotes the participant's response, ^2 ^***I: ***Denotes the interviewer's questioning)

Manufacturer #1 also focuses on those making/influencing purchasing decisions, which includes management and administrative staff. Manufacturer #1 articulates this shift in focus when asked who they view as their customer:

**#1**: "Orthopaedic surgeons really. Although it is used in patients, it is the orthopaedic surgeons who decide what he will use. Sell to orthopaedic surgeons. Increasingly in the UK, we have to sell to the hospital management to justify why they should be using our bone graft substitutes as opposed to any other on the market..."

Manufacturer #8 stated that health professionals are considered to be the main user of their device, However, the opinions of purchasing representatives are the primary source used to inform device design and development, followed up by consultation with a small number of 'clinical champions' (typically high profile surgeons and well known experts in their field). When questioned on what value the user may add value to the MDDD process, although couched in a humorous reply, it was clear that the usefulness of the user was ideally directly located in relation to sales relevant information.

**#8**: "The most helpful would to give me the year three sales figures with absolute confidence. Year-1...[laughs]..."

There seems to be limited overlap between the individuals that will be using the device, and those that are consulted to inform the MDDD process. In particular, priority is given to those that hold more senior positions within the health care system. Therefore, surgeons, doctors and clinical champions were seen as more valuable sources for identifying user needs as opposed to those individual that would actually use the device on a daily basis. Furthermore, from the manufacturers' point of view, the motivation for maximising sales seems to have conflated the distinction between the customer and the user. Therefore the needs of those that make purchasing decisions or indeed have most influence over these decisions are more salient than the needs of the user. This is certainly a more complex picture than the human factors engineering approach, which puts the user's needs at the centre of the design and development process.

### Methods used

Given the wide range of formal methods that are available to engage with users in the MDDD process, manufacturers tended to use only a very limited range of methods to capture information from users and patients. In line with the analysis above regarding the nature of the preferred user to consult, the most typical method used to gain input from users was via informal discussions with senior health professionals. Only one out of the eleven manufacturers (Manufacturer #8) stated that they regularly used some formal methods throughout the MDDD process, such as focus groups and questionnaires when developing their airway management device. Interestingly, the initial identification of a need for this device occurred as a result of six members of the company attending a postgraduate university course, which required the use of formal methods in order to explore and identify new device ideas. It was apparent that manufacturers do not feel that they have the time or resources to engage in rigorous formal user data collection methods, instead relying on a range of strategies including gut feel, instinct, and a personal belief that they understand the market place in which they operate, in order to identify and develop new devices.

The idea of employing formal methods is once again something that is not regarded as feasible, given the amount of resources available and the fact that manufacturers believe it is necessary to move quickly in order to remain competitive with their rivals. The view is that consulting a large number of individuals is problematic in itself, as every person that is approached for feedback has a different view. Informal methods, however, are seen as offering versatile and rapid solutions, hence the belief that relying on gut feel and pressing ahead when the moment feels right is a more feasible and efficient solution. This point is articulated by Manufacturer #8 below:

**#8**: "The very fact that someone is willing to talk with you almost means that they have slightly different view. You can go on asking forever. It's that balance between have we got sufficient confidence in what we have here to move forward vs. just the generation of the information....being confident enough of its assurance. That's what you constantly face anyhow. I think that's the dilemma you always have. You can ask the users till the cows come home but you never get a new product. You ask 100,000 you get 99,999 different opinions!"

Manufacturer #3 further echoes the observation that formal methods are rarely used for medical device development, however, informal discussions and observation are seen as more versatile and fit for purpose, :

**#3: P**:"you're introducing a medical device to people, first question they'll ask, is "what will it do to help me?" Second question will be "how long is this going to take?" In a busy clinic that's really important...If it takes too many clicks, then people won't use it because they are busy enough as it is."

**I**: have you been able to capture that at all?

**P: "**Through our interaction with users. We haven't got a specific mechanism for capturing it."

**I**: Do you use any formal methods for converting customer needs into product development?

**P**: "No."

There was typically the belief that there was little need to consult the actual users formally regarding a new innovation, but rather contacting what they referred to as a 'clinical champion', was sufficient to qualify the feasibility and validity of a given new innovation. For example, Manufacturer #8, responding to a question regarding how the feasibility of new device ideas are qualified responded:

**#8**: *"Every project will have a clinical champion. They will typically be involved, sometimes they come down here to meetings. We have a list of a couple of dozen clinicians that I can pick the phone up at anytime throughout the world and say "what do you think of this?".*

When asked whether there are any formal methods used within their organisation, Manufacturer #6 stated that formal methods are used within his organisation, however, they are not relevant for this particular product. Once again, formal methods, in this case example, were considered to be too bureaucratic, time consuming, and not applicable given the device and development scenario. Informal methods, such as ad hoc discussions with senior health professionals were likely to identify the majority of user design needs, and were also more appropriate.

**#6:"**Yes we do, but I couldn't apply that to hip replacement at this point in time. If the surgeon tells me "that this catheter is a bit too stiff, and could you make it a bit softer, if you like, or a bit more flexible, yet do exactly the same task?" we will do that - it's for everyone's benefit"

In the above example, a pragmatic approach is taken by manufacturers to make modifications to devices, adopting the belief that if a problem is encountered by one individual, then it is likely to be a problem for the majority, providing it seems to be a reasonable request. The intuition of the manufacturer plays a major part in the process of developing products that are useful to the user, and responding to their needs.

### Perceived value and barriers to user involvement

There was limited evidence that direct elicitation of user views was seen by manufacturers as being of value to the MDDD process. This appeared to be particularly the case in relation to patient users. For example, Manufacturer #11, discussing their device that was purely aimed at the home patient market, did not believe that patient involvement in the MDDD process was a particularly wise expenditure of resources. This was explicitly linked to the degree of influence that they have in terms of the level of power and influence they have in the levels of uptake of the device. In response to being asked whether they would like to involve the patient more in the MDDD process, manufacturer #11 replied:

***#11: **"if they are highly powered (i.e. influential in terms of clinical decision making), if they have no power then we have to ethically try to make sure that we don't harm the patient and [that] *what *we do is for their benefit, but appealing to them may actually be a waste of resources, so we have to make sure we don't pretend that they have a sway when they really don't, you know? "*

Once again, the above statement reinforces the notion that patients are seen as being at the bottom of the hierarchy of influence, and hence investing resources and effort to find out their opinion is not considered an effective of efficient strategy. Manufacturer #2, who also saw their device as being targeted at home market, acknowledged that the patient user is becoming increasingly important, particularly as they are now selling more directly to the home patient. However, the majority of individuals involved in the MDDD process are still predominantly senior health professionals as opposed to patients, who may play a stronger role in the clinical trials phase, after the device has been designed and developed. Therefore, the role of the patient user is considered to play a passive role, more in terms of verifying the value of an already developed device, as opposed to being involved in the initial design and development stages. This is discussed in more detail in the next section.

Another factor discouraging manufacturers from involving patient users in the MDDD process is the prospect of having to obtain ethical approval in order to carry out the research. When asked whether there are any difficulties in obtaining ethical approval for involving patient users in the MDDD process, Manufacturer #3 stated:

**#3**: "Yes. A huge problem. In my experience if you are not affecting patient care, patient throughput, you can get 'ethics', but if you are you won't get it, or you might but it will take years. So you have to design your trial so as not to affect patient care."

Manufacturer #1 also comments on the ethical approval process and the R&D committees that must be attended to when employing more formal methods users, both patient users and professional users in the collection of clinical data.

**#1**: R&D committees. That's another thing. You start a study and surgeon wants to collect clinical data to be sure that they want to use this. Their experience that this is the product they want to use. You have to notify the hospital R&D and then suddenly they see it as an R&D project, so they throw on their overheads and it's an extra hurdle to go through. They start reviewing it in addition to ethical board. This is what has changed in the last few years. In those days you notified the larger University hospitals. Increasingly has to be approved. Some are straightforward; others ask extra questions that delays process - UK research.

This perceived difficulty with obtaining ethical approval may be one reason why manufacturers tend not to use formal methods, or engage in systematic research activity in order to inform the MDDD process, particularly at the early stages of development. Indeed manufacturers appear to actively avoid involving professional and patient users in the process, in fear that the MDDD process could be delayed for years as a result of the ethical approval process. As identified in section 3.2, informal discussion with clinical staff is seen as a more realistic, pragmatic, and feasible route to informing the MDDD process, which can be carried out informally and hence without ethical clearance. Manufacturer #3 goes on to describe the strategy that they use to get around the challenge of obtaining ethical approval:

**#3**: "*So you take the least hard route, to not affect patient care in any way whatsoever, and design your study to sit on the back of that. So the study might not be optimal, but at least you can do it."*

Setting up device design and development activity so as to avoid the need for user involvement seems to be the 'method' of choice for some device manufacturers. Despite this being seen as a potentially sub-optimal approach, perhaps less effective in evaluating the extent to which design innovations may be accepted by users, it seems a route worth taking given the alternative of incurring long delays as a result of the ethical approval process.

### The nature of user contributions

The measures used to evaluate the effectiveness of newly developed devices towards the end of the MDDD process echo the findings presented earlier, that formal methods employed in any part of the MDDD process are seen by manufacturers as having the potential of slowing the process down, and incurring additional and unnecessary costs and overheads that otherwise could be avoided if a less formal approach was taken. Most commonly, manufacturers reported that the success of a new device is typically measured by the absence of receiving customer complaints or 'bad news' emerging as a result of the device being used in the field. Manufacturer #1, in response to being asked how the success of their device is measured responded as follows:

**#1**: "The absence of bad news. The fact that we have access to the surgeons who are using the product and any adverse effects would be reported and we would be aware early on."

With regards to seeking patient feedback about the success of a medical device in providing an effective health intervention, Manufacturer #3 takes a similar 'no bad news is good news' approach to the design and functioning of their product. When asked whether any patient feedback is sought about the device, the reply was:

**#3**: "*Not explicitly, we haven't gone out to get it, but we get feedback though the users (clinicians). It's non-invasive, so as far as the patients concerned it's not a problem to use it. No-one has said they don't want it done or had any problems with that."*

Manufacturer #9, commenting on whether any formal methods are used for converting user needs into device design requirements, also indicated that they adopt a reactive stance to customer suggestions and complaints, which is their default position on such matters.

**#9**: "Erm, we certainly have regular meetings here where we will look at customer feedback, you know, whether it's customer complaints or customer suggestions or whatever, yes so, yes we do have a means to do that..."

Some evidence of formal user involvement did emerge from the interview data, however, it indicated that the majority of formal user involvement took place at the clinical trials stage, i.e. after the product had been developed, and manufacturers were at the stage of demonstrating its efficacy and clinically effectiveness. Therefore, in effect any considerations in terms of design preference of the medical device that may benefit the user in terms of their treatment or use of the device had already been made prior to this formal user involvement. Manufacturer #2 highlights the notion that the patient user is primarily seen as being of value when attempting to demonstrate the clinical effectiveness of a new medical device.. Interestingly, patient users are not seen as primarily informing the general design of the device at earlier stages of the MDDD process, at least not in a formal capacity. When asked whether formal user methods are employed within the MDDD process, Manufacturer #2 responded:

**#2**: "There is the formal method of gaining patient data which means at the moment for example we have conducted clinical trials, clinical investigations. All sorts of clinical information gathering is going on at the [hospital name]. We are also... we tend to gather information first of all on the effectiveness or efficacy of it...Any advance, any variation that is likely to happen to the unit, any proposals for change that are going to improve the machine are all tested out in the clinical environment."

Manufacturer #5 reported that the key driver for collecting formal user data, relating to the performance of a device, comes from the possibility that an organisation such as the National Institute for Clinical Excellence (NICE) may decide to investigate the efficacy of their device at some unknown point. Therefore, the key driver for formally collecting user generated data is to fulfil the potential future requirements of external standards or purchasing agencies. The motivation to collect user facing data does not appear to be borne out of an inherent belief, on the manufacturer's behalf, that this user data would add any significant value in terms of fulfilling their own need to develop more effective devices or indeed learn more about the effectiveness and efficiency of their own device.

**#5**: I think, if you take an organisation like NICE as a customer, or any of the other health technology organisations, one doesn't know whether your particular intervention is going to be assessed by them, and if so at what point, and one can see that as [the product] gains momentum and the NHS starts to look at what its spending on [this] surgery, then it may be something that NICE feel, or NICE get directed to take a look at. So it's a kind of a problem to us to understand when that's going to happen and it will certainly be a challenge; we have to therefore make sure that we are gathering the evidence in case they do."

## Discussion and Conclusions

In this study, 11in-depth interviews were carried out with medical device manufacturers, who were asked to comment on the role and value they believed that users have within the MDDD process. Given the small sample size, it is recognised that the results of this study should be considered as provisional. However, given the limited existing research in this area, the findings provide an important point of reference for further work.

The results revealed that manufacturers tend to prioritise the views of more senior health professionals above those that are less senior as well as patients. Furthermore, manufacturers' perceptions of the customer and the user has become conflated, partly due to the strong sales focus of manufacturers, seeking device design input from those individuals who make purchasing decisions, as opposed to the users of the devices. With regards to seeking input from the patient user, there was little motivation to engage in such practice which was seen as an ineffective use of resources, with patients, and less senior health professionals being perceived as having little impact or influence on general device sales, largely due to the inbuilt culture of patients being 'taught down' their needs from health care professionals.

Only one out of 11 manufacturers claimed to regularly use formal user centred design methods within the MDDD process. Interestingly, this individual also reported that they were familiar with such methods as a result of being introduced to them within the university setting in which they carried out the initial formulation and design of the product they were discussing. This experience appeared to have had a lasting effect, as they report the ongoing use of formal research methods to this day. It is therefore possible that a contributory factor to lack of engagement with formal methods is a lack of education, familiarity or confidence in their use. Necessary training has indeed been found to be a factor that affects the level of uptake of usability methods in medical device development [34]. Informal methods were typically preferred by manufacturers, in the form of extemporised discussions with a small number of esteemed medical experts. There was also a belief that if a manufacturer wished to be competitive and responsive within a fast moving market, formal methods do not offer the necessary versatility and are more generally not appropriate for purpose, despite the proposed benefits that academic literature suggests such methods promise if applied within the health care sector [35,36]. Research in the technology design domain suggests it is necessary to adopt a flexible and evolutionary stance when applying formal methods to cater for the unique context and organisational cultures that present themselves within any new design challenge [37]. Similarly, there may be value in increasing awareness of the versatility of existing human factors engineering methods, and in specialist contexts to explore the potential development of more agile and tailored methods that cater for manufacturers' needs in the context in which they are to be applied. It has been suggested that, as a consequence of the increased pressure from standards agencies to incorporate formal user methods into the MDDD process, some manufacturers may 'misuse' existing human factors methods [38]. The reasons for this are unclear. Is it perhaps in a bid to make methods more fit for purpose, or as a result of not being fully aware of the ways in which existing methods should be applied?

The perceived barriers to user involvement within the MDDD process were linked to the notion that manufacturers seek out those individuals that will be most influential in making purchasing decisions for their products. Consequently, involving patient users appeared to be of lowest priority, since it was believed that they held the lowest level of influence over whether health care organisations purchased their products. These findings are supported by our earlier preliminary work relating to barriers to user involvement [39,40]. The examples that did emerge of consulting users via formal methods, tended to occur at the end of the MDDD process, after the device had been designed and developed, where users played a passive role in aiding the manufacturer in verifying the efficacy of the device primarily for the purposes of satisfying external standards and purchasing agencies requirements. One key reason for avoiding user involvement was the difficulty that this presents when attempting to gain ethical approval for user elicitation studies. Manufacturers conceded that, although avoiding user involvement may perhaps be sub-optimal, it is necessary if they are to remain competitive within a fast moving market. Warlow [41] and Stewart et al. [42] both propose that over-regulation of clinical research poses a significant threat to public health and it seems that this study support this. In particular, our findings suggest that the seemingly unnecessary bureaucracy associated with obtaining ethical approval for non-interventional and low-risk studies, that seek to capture user opinions and requirements, leads to manufacturers excluding the voice of the user from the development process.

This study reveals that the notion of proactively involving the user within the MDDD process in general slows down the process. Rather, a reactive 'no bad news is good news' stance is taken, only taking into account users input if they are presented in the form of complaints or feedback on devices that have already been released into the health care system. The only evidence of engagement with formal methods of user involvement is apparent when the use of such methods are mandatory, dictated to manufacturers by standards and purchasing agencies. Given the findings of this study, the appropriate employment of formal methods by manufacturers is unlikely to occur to significant levels without deliberate efforts to encourage and support manufacturers in doing so. The following recommendations propose where some of these efforts should focus, in order to achieve increased levels of user engagement by manufacturers, as is now stipulated by IEC 62366 [14]:

- Research to better understand the requirements of manufacturers, in terms what is required from human factors engineering methods in order to make their use more feasible and accessible in practice.

- Provision of training on the use and benefits of employing formal human factors engineering methods at every stage of the MDDD process.

- Health care providers should implement formal processes to ensure better communication and synergy between those making purchasing decisions and the actual users of the devices.

- Provisions should be made within the ethical approval process that enables medical device manufacturers to engage more easily with users with minimal levels of bureaucracy whilst also ensuring that all research is conducted in an ethical manner that protects healthcare staff and patients.

## Competing interests

The authors declare that they have no competing interests.

## Authors' contributions

All authors contributed to the conceptual design of this study. AGM and JB carried out primary data analysis and drafted the manuscript. MPC and JLM contributed to data collection and provided domain expertise. All authors contributed to redrafting the manuscript.

## Pre-publication history

The pre-publication history for this paper can be accessed here:

http://www.biomedcentral.com/1472-6947/11/15/prepub
